# Molecular profiling of advanced solid tumours. The impact of experimental molecular-matched therapies on cancer patient outcomes in early-phase trials: the MAST study

**DOI:** 10.1038/s41416-021-01502-x

**Published:** 2021-09-07

**Authors:** Valentina Gambardella, Pasquale Lombardi, Juan Antonio Carbonell-Asins, Noelia Tarazona, Juan Miguel Cejalvo, Inés González-Barrallo, Jorge Martín-Arana, Roberto Tébar-Martínez, Alba Viala, Gema Bruixola, Cristina Hernando, Inma Blasco, Federica Papaccio, Carolina Martínez-Ciarpaglini, Clara Alfaro-Cervelló, Enrique Seda-García, Sebastián Blesa, Isabel Chirivella, Josefa Castillo, José Vicente Montón-Bueno, Susana Roselló, Marisol Huerta, Alejandro Pérez-Fidalgo, Paloma Martín-Martorell, Amelia Insa-Mollá, Tania Fleitas, Pilar Rentero-Garrido, Sheila Zúñiga-Trejos, Andrés Cervantes, Desamparados Roda

**Affiliations:** 1 Department of Medical Oncology, Hospital Clínico Universitario de Valencia, INCLIVA Biomedical Research Institute, University of Valencia, Valencia, Spain; 2 CIBERONC, Instituto de Salud Carlos III, Madrid, Spain; 3 Division of Medical Oncology, Institute for Cancer Research and Treatment, IRCCS Candiolo, Candiolo, Italy; 4 Bioinformatic and Biostatistic Unit, INCLIVA Biomedical Research Institute, Valencia, Spain; 5 Precision Medicine Unit, INCLIVA Biomedical Research Institute, University of Valencia, Valencia, Spain; 6 Translational Oncology Unit, INCLIVA Biomedical Research Institute, University of Valencia, Valencia, Spain; 7 Department of Medicine, Surgery and Dentistry “Scuola Medica Salernitana”, University of Salerno, Baronissi, SA Italy; 8 Department of Pathology, Hospital Clínico Universitario de Valencia, INCLIVA Biomedical Research Institute, University of Valencia, Valencia, Spain; 9 Department of Biochemistry and Molecular Biology, University of Valencia, Valencia, Spain

**Keywords:** Molecular medicine, Tumour biomarkers

## Abstract

**Introduction:**

Molecular-matched therapies have revolutionized cancer treatment. We evaluated the improvement in clinical outcomes of applying an in-house customized Next Generation Sequencing panel in a single institution.

**Methods:**

Patients with advanced solid tumors were molecularly selected to receive a molecular-matched treatment into early phase clinical trials versus best investigators choice, according to the evaluation of a multidisciplinary molecular tumor board. The primary endpoint was progression-free survival (PFS) assessed by the ratio of patients presenting 1.3-fold longer PFS on matched therapy (PFS2) than with prior therapy (PFS1).

**Results:**

Of a total of 231 molecularly screened patients, 87 were eligible for analysis. Patients who received matched therapy had a higher median PFS2 (6.47 months; 95% CI, 2.24–14.43) compared to those who received standard therapy (2.76 months; 95% CI, 2.14–3.91, Log-rank *p* = 0.022). The proportion of patients with a PFS2/PFS1 ratio over 1.3 was significantly higher in the experimental arm (0.33 vs 0.08; *p* = 0.008).

**Discussion:**

We demonstrate the pivotal role of the institutional molecular tumor board in evaluating the results of a customized NGS panel. This process optimizes the selection of available therapies, improving disease control. Prospective randomized trials are needed to confirm this approach and open the door to expanded drug access.

## Background

During the past few decades, cancer treatment has been revolutionised by the discovery of molecular aberrations recognised as major drivers of cancer initiation and progression [1]. This remarkable advance in molecular understanding was followed by the emergence of a new oncology paradigm called precision medicine. Personalised cancer medicine has the main aim of finding a selective targeted therapy for the specific tumoral molecular alterations found in each individual tumour. The capability to inhibit specific molecular targets has already improved the clinical outcomes, becoming the standard of care in several solid tumours, such as breast [2–4], lung [5–7] colorectal cancers and melanoma [8, 9]. Nevertheless, precision medicine still presents several limitations such as intrinsic cancer heterogeneity [10, 11], the complexity of the tumour microenvironment [12–14] and evolving molecular clonal dynamics [11] as mechanisms of treatment resistance.

Several prospective and retrospective studies have been conducted to investigate the benefit of targeting molecular aberrations (characterised using next-generation sequencing (NGS) or transcriptomic analysis) for a precision approach in the non-restricted histology population [15–18]. Interestingly, some trials were positive, whereas others were surprisingly negative, showing the intrinsic limitations of inhibiting a single molecular target and the complexity of cancer biology.

Early (phases I and II) trials are designed to evaluate the tolerability of a new agent and identify the recommended doses for further clinical development, as well as to evaluate preliminary signs of antitumor response. Phase I clinical trials have historically been considered of low clinical utility [8]. Nevertheless, as molecular-based medicine has evolved, and new predictive drivers have been identified, it is not uncommon to see significant antitumor activity when targeted agents are tested on molecularly selected populations in early drug development. This has been confirmed in several specific cases with promising results, such as the ones obtained in early-phase trials, with crizotinib [19] or with the first-generation NTRK inhibitors [20, 21] larotrectinib and entrectinib. The clinical benefit obtained led to tumour-agnostic regulatory approvals for the treatment of EMLA4/ALK-translocated NSCLC and NTRK gene fusion-positive solid tumours.

In the currently evolving landscape of early clinical trials, biomarker-based patient selection studies may represent a valuable therapeutic option. Here, we report the results from a single-centre, retrospective analysis, where we investigated the feasibility and efficacy of comprehensive molecular profiling in patients with advanced solid tumours. Our first aim was to explore the benefit of a molecular profiling selection approach for early trial candidates in the context of targeted agents. Second, we investigated the impact of those targeted therapies vs standard therapies in subsequent treatment options for our advanced cancer patients. Third, using a Bayesian model approach, we sought to define the primary predictive factors for potential treatment benefit.

## Methods

### Patient selection

Eligible patients were aged ≥18 years, had advanced solid tumours, Eastern Cooperative Oncology Group (ECOG) performance status ≤2, tissue available for molecular testing, a life expectancy of at least 12 weeks and were likely to meet the additional enrolment criteria specified in each trial protocol. All tumour samples were locally studied and analysed for individual genomic target alterations by NGS, evaluating DNA. In those cases, in which the transcriptomic analysis could have helped in treatment options, an RNA analysis was also implemented. Some samples were submitted for central evaluation if required by the study protocol. Patients enrolled in the clinical trials signed the specific consent accordingly.

### Sample selection

As per the protocol, tissue analysis was performed in our certified laboratory. All tumour samples analysed were formalin-fixed paraffin-embedded (FFPE). Verification of adequate tumour cellularity (at least 20%) was performed by board-certified pathologists.

### Targeted sequencing

Genomic analysis was performed by a customised panel that included the evaluation of hotspot mutations in 83 genes + 4 full genes analysed. The panel captured a total of 43.2 kb. This panel has been validated internally following the guidelines of the Association for Molecular Pathology and College of American Pathologists [22]. For validation, HapMap cell lines NA12878 and NA1277 were used along with Horizon Discovery controls (HD734 and HD827). Using these control samples, we determined our limit of detection as a 5% variant allele frequency (VAF) in samples with a percentage of tumour cellularity >20% and >20 ng/μL of DNA. The positive percentage agreement (PPA) and the positive predictive value (PPV) was 100% and 92.4%, respectively. In addition, the Precision Medicine Laboratory participate in the 2019 Oncogene Panel scheme of the European Molecular Quality Network as an interlaboratory assessment with satisfactory results for this panel. Samples were extracted using the Maxwell(R) 16 FFPE Plus LEV DNA Purification Kit (Promega). DNA-sequencing libraries were prepared using KAPA HyperPlus Kit (Roche Diagnostics). Extracted DNA was enzymatically fragmented, and after end repairing and A tailing, sequencing adaptors were ligated onto both ends. Targeted enrichment was performed using the SeqCapEZ Prime Choice Probes (Roche Diagnostics) whose content was the customised panel and the HyperCap Target Enrichment Kit (Roche Diagnostics) to amplify the captured regions. Targeted libraries were sequenced in a MiSeq sequencer platform (Illumina Inc.) with MiSeq v2 300 Cycles Kit, following the manufacturer’s instructions.

Transcriptomic protocol for fusion analysis was performed by RNA sequencing with ribosomic depletion. For validation, 5-Fusion Multiplex-HD784 and 5-Fusion Multiplex Negative Control-HD783 and previous samples characterised by fluorescence in situ hybridisation were used; the PPA and PPV were 98% and 95%; respectively. The tumour samples selected for the transcriptomic analysis has at least 20% tumour cellularity, and RNAs were isolated using Maxwell 16 FFPE LEV RNA Purification Kit (Promega). RNA-sequencing libraries were obtained using the KAPA RNA Library Prep + Riboerase Kit (Roche Diagnostics) and sequenced in a NextSeq sequencer platform (Illumina Inc.) with NextSeq v2.5 HighOutput 300 Cycles Kit, following the manufacturer’s instructions.

### Next-generation sequencing analysis

Data analysis was carried out using an in-house-developed bioinformatics pipeline. Briefly, raw data quality was assessed with FASTQC [23]. Low-quality reads were filtered out with PrinSeq [24], applying an average read quality score of 30. Read mapping was performed against the latest human reference genome (GRCh38) with the Burrows-Wheeler Aligner (BWA-MEM algorithm) [22]. Read alignment transformations, duplicate read removal and sample enrichment assessment at different read depths were performed with Samtools [25], PicardTools (http://broadinstitute.github.io/picard) and BedTools [26], respectively. Variant calling was performed using a combination of two different tools, Mutect2 [27] and LoFreq [28]. Annotation was performed with SnpSift [29] and the Variant Effect Predictor tool from Ensembl [30]. Pathological somatic variants were identified by querying our cancer mutation database, which contains verified information from different resources such as PCT MD Anderson (https://pct.mdanderson.org), commercial panels (Oncomine® and Sequenom®), the cancer hotspot database (www.cancerhotspots.org), relevant literature and our own expertise.

All samples obtained a mean coverage of 250×. Genetic variants with VAF >5% identified in the patients were clinically evaluated using several public databases (ClinVar [31], Varsome [32], OncoKB [33] and cBioPortal [34]). Only variants classified as pathogenic, likely pathogenic and uncertain significance were reported.

### Genomic analysis and treatment selection

**Fig. 1:**
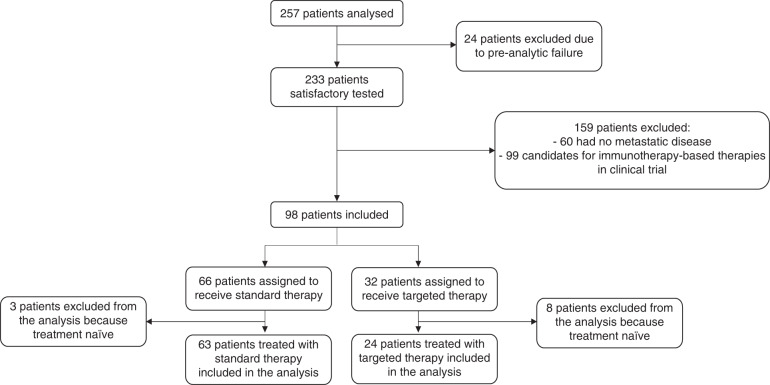
Consort diagram. The flowchart showes the subject enrollment and treatment allocation.

Each individual genomic report available was reviewed and discussed weekly by a multidisciplinary tumour board dedicated to precision medicine, attended by experts in clinical oncology, molecular biology, pathology, clinical genetics and bioinformatics. Actionable targets were defined by the tumour board according to the existing level of evidence [35, 36], and molecular-based treatment suggestions were proposed where possible. (Fig. 1).

Individual patient therapy options were discussed by the tumour board [37, 38] using the following decision strategy: (i) treatment targeting the identified driver alteration in an early trial; (ii) treatment targeting the proposed driver alteration as compassionate use; (iii) if no treatment-relevant driver mechanism was found, the best clinical choice was proposed. Patients harbouring a molecular aberration received specific experimental therapy based on the tumour board recommendations in the setting of phase I–II trials [39, 40]. The response was assessed with scheduled disease evaluations every 6–8 weeks, according to RECIST1.1 [41]. It is noteworthy that all patients diagnosed with a cancer type for which genomic testing is part of the standard of care (lung cancer, gastrointestinal cancer, breast cancer, etc.) were previously tested and treated according to standard guidelines and were not considered for this analysis.

### Statistical analysis

Patient characteristics have been described according to variable type, continuous variables as median with its interquartile range, and qualitative variables with frequencies and percentages. Outcome variables included progression-free survival one (PFS1) of the previous line of therapy given immediately before molecular profile results were available; progression-free survival two (PFS2) of the experimental vs standard therapy after genomic results were available. PFS ratio was measured as the ratio of PFS2 vs PFS1 (immediate prior line of therapy), that is, using patients as their own control. PFS (PFS1, PFS2 or PFS2/PFS1) was only evaluated on patients who had a prior treatment line. Kaplan–Meier curves were applied to compare the survival of patients that underwent molecular profiling vs non-targeted therapy for the first and second line to test for prior selection bias. Pairwise survival Bonferroni-adjusted test was used to compare treatment type and diagnosis (breast cancer vs other). Multivariable Cox regression model for PFS2 was applied with treatment type as an independent factor and adjusted for age, previous treatment (yes or no), performance status (PS), leucocyte, lymphocytes, neutrophil, Royal Marsden Hospital (RMH) Prognostic Score and interaction between previous treatment and treatment type. PFS ratio was dichotomised with a cutoff of 1.3 as in previous molecular-matched trials [17]. Fisher exact test was used to evaluate the relationship between treatment type and PFS ratio and tested to ascertain whether the proportion of patients who underwent targeted therapy and had a PFS ratio >1.3 was >0.15.

Bayesian model averaging (BMA) was carried out to select the best model according to the posterior probability for dichotomised PFS ratio as the dependent variable, and age, PS, treatment, leucocyte, lymphocytes, neutrophil and RMH as independent variables. We assume a Bernoulli distribution for the dependent variable *R*_*i*_, which has a value of 1 for patients with a PFS ratio >1.3 and 0 otherwise. The proposed likelihood for our model is:

$$R_i\sim {{Bernoulli}}(\pi _i)\quad \quad i = 1, \ldots ,87,$$

$${{logit}}(\pi _i) =	\, \beta _0 + \beta _1{{Age}} + \beta _2{{PS}} + \beta _3{{Treatment}} + \beta _4{{Leucocytes}}\\ \!\!	+\, \beta _5{{Lymphocytes}}\ + \beta _6{{Neutrophil}} + \beta _7{{RMH}}.$$

The prior distribution of our parameters is based on a non-informative Normal distribution as:

$$\beta _k = {{Normal}}\left( {0,\,10,000} \right)\quad \quad k = 0, \ldots ,7$$

Convergence will be accepted if the Gelman–Rubin statistic is ≤1.1 and posterior parameters’ effective size is >100 [42].

All results in the frequentist approach will be considered statistically significant if *p* < 0.05. Bayesian results for posterior parameter distribution are presented with mean, standard deviation and credibility confidence interval (CI). All analyses were carried out with R version 3.6.2 [43], BMA with BMA package [44] and Bayesian best model with WinBUGS [45] and R2WinBUGS [46].

## Results

### Patient characteristics

A total of 561 patients diagnosed with advanced solid malignancies were referred to our INCLIVA Precision Medicine Unit from January 2019 to April 2020 for potential inclusion in early-phase clinical trials. Two hundred and fifty-seven patients were tested with our customised hotspot NGS panel, while 25 patients were analysed centrally with an NGS transcriptomic panel, according to the specific trial protocol in which they were included. Of the 257 patients admitted to our molecular screening programme to be analysed, after a pre-analytic evaluation, 231 patients were satisfactorily tested. One hundred and thirty-three patients were excluded from this analysis: 60 were not metastatic and were enrolled into the clinical trial for adjuvant/neoadjuvant treatment, while the others were considered eligible for immunotherapy-based therapies. A total of 98 patients were finally included, 87 of which had a prior treatment line (Fig. 1).

Patient demographic characteristics were similar in the enrolled vs non-enrolled groups. The main patient characteristics of the 98 patients included are reported in Table 1.

**Table 1:** Patient characteristics.

	All patients	Targeted therapy	Standard therapy	*P* value
Population	98	32	66	<0.001^a^
Median age, range	61, 24–83	59, 25–81	62, 24–83	0.507^b^
Sex				
Male (*n*, %)	32 (33.7)	6 (15.6)	27 (40.9)	0.040^c^
Female (*n*, %)	66 (67.3)	27 (84.4)	39 (59.1)	0.023^d^
ECOG PS				
0	23	12	11	0.043^d^
1	61	19	42	0.853^d^
2	14	1	13	0.032^c^
Tumour type				
Breast	22	12	10	0.026^d^
Gynaecological	25	10	15	0.509^d^
Lung	22	3	19	0.039^c^
Digestive	15	5	10	1.000^c^
Head and Neck	8	1	7	0.267^c^
Skin	2	1	1	0.549^c^
Others	4	0	4	0.300^c^
RMH prognostic score				
Good	73	23	50	0.868^d^
Poor	23	8	15	1.000^d^
Missing	2	1	1	0.549^c^
Previous lines (median)	1	1	1	1.000^b^

*ECOG PS* Eastern Cooperative Oncology Group Performance Status, *RMH prognostic score* Royal Marsden Hospital Prognostic Score.

*P* value are calculated as follows: ^a^Binomial test, ^b^Kruskall–Wallis test, ^c^Fisher’s exact test and ^d^two-sample proportions *z* test.

The most common tumour types were gynaecological (*n* = 25), lung (*n* =  22), breast (*n* = 22), digestive (*n* = 15) and head and neck cancers (*n* = 8). In the enrolled subjects, based on an actionable mutation group, the median age was 61.1 years, patients were predominantly female (67.3%), had received a median of one prior systemic treatment and had a median ECOG performance status (PS) of 1. The Royal Marsden Hospital (RMH) score was 0–1 in the majority of patients (74,5%).

### Molecular results

**Fig. 2:**
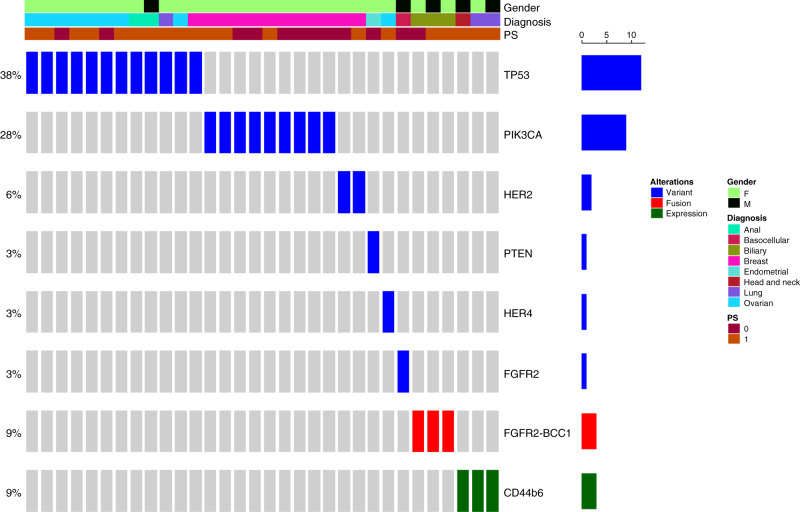
Most common genomic alterations detected with our in-house NGS customised panel. This graph shows the percentage (left) and absolute frequency (right) of genomic alterations detected across the study subjects. NGS next-generation sequencing.

Druggable molecular alterations are reported in Fig. 2. An actionable target was detected in 32 patients (32.6%). An alteration of the DNA repair response pathway was detected in 38% of patients, followed by PIK3CA mutations (28%), FGFR alterations (FGFR2 mutations 3% FGFR gene fusion 9%), MET activations (9.4%), ERBB family pathway alterations (ERBB2 6%, ERBB4 3%) and PTEN mutations (3.1%) (Supplementary Tables 1 and 2).

**Fig. 3:**
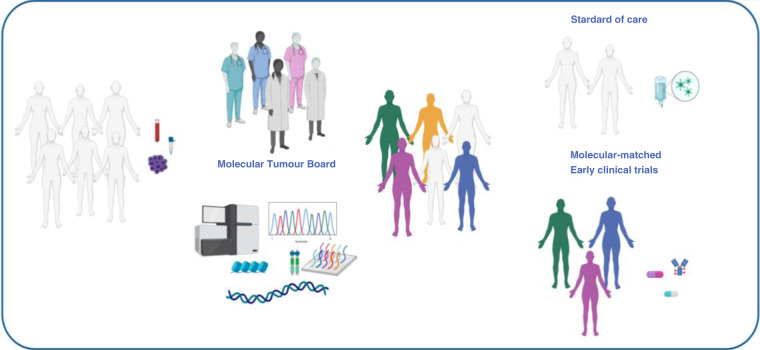
Schematic representation of our Molecular Tumour Board. The picture summarizes the process which leads to the identification of new potential candidates for experimental therapies based on molecular analysis.

All molecular findings were discussed on our weekly molecular tumour board, with patients subsequently directed to molecular-driven trials, novel immunotherapeutic strategies or standard of care according to molecular alterations and the inclusion criteria of each single trial (Fig. 3). Specifically, patients were mostly included in specific protocols developing novel PIK3CA, AKT, FGFR, Check-1, MET, HER2 and PARP inhibitors with novel indications.

### Antitumor activity

**Fig. 4:**
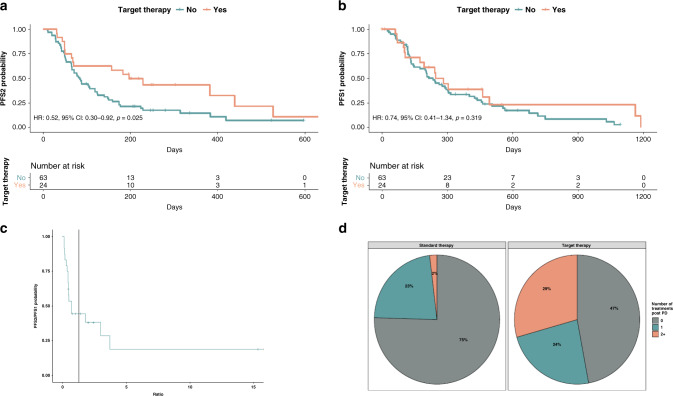
Outcome analyses of the included population: PFS2, PFS1, PFS2/PFS1 ratio and further lines beyond progression. **a** Progression-free survival after inclusion in the trial (PFS2) was compared for molecular-matched therapy vs standard of care. **b** PFS derived from the previous therapy for the patients into the two groups. **c** PFS2/PFS1 ratio in the experimental arm with a vertical line showing 1.3 as the cutoff. **d** Number of further treatment lines received by the patients beyond progression in the two groups.

PFS2 was compared for molecular-matched vs unmatched patients (Fig. 4a). Patients enrolled in early-phase trials developing novel immunotherapies were excluded from this analysis, as were those treated in the first line. Patients receiving matched therapy according to their molecular profile had a higher median PFS2 (6.47 months; 95% CI, 2.24–14.43) than patients whose therapy was not matched (2.76 months; 95% CI, 2.14–3.91, log-rank *p* = 0.022). Interestingly, no significant differences were found comparing median PFS1 with their immediately previous therapies between patients treated with targeted vs non-targeted therapy (PFS1) (*p* = 0.56), (Fig. 4b).

The proportion of patients in the molecularly matched group with a PFS2/PFS1 ratio ≥1.3 was 33% (8 out of 24; 95% CI, 0.16–0.55), while only 7.9% (5 out of 63; 95% CI; 0.01–0.14) in the control arm had reached that threshold (*p* = 0.026). The association between treatment type and PFS ratio was also significant according to Fisher’s exact test (*p* = 0.005). Figure 4c shows the survival curve of patients in the experimental arm with a vertical line indicating the 1.3 ratio cutoff.

All patients enrolled were evaluable for response assessment according to RECIST1.1. In total, 63.6% of patients treated with standard therapy experienced disease progression (PD) as the best response, vs 31.3% of those enrolled into the experimental arm (*p* = 0.005).

### Multivariable analysis

In multivariable analysis, treatment type (molecular-matched vs standard), low lymphocyte count, high leucocyte count, RMH prognostic score and ECOG PS were the only statistically significant factors independently associated with prolonged PFS2 (Supplementary Table 3).

In order to minimise confounding factors, a contingency analysis was performed, excluding breast cancer patients in both subgroups. Patients who received matched therapy after their molecular profiling results had a higher median PFS (6.48 months; 95% CI, 2.24–14.43) than patients who received standard therapy (2.33 months; 95% CI, 1.74–3.45; log-rank *p* = 0.034). Subsequently, a subgroup analysis in which four different cohorts were analysed (breast-targeted, breast-no targeted, other solid tumour-targeted, other solid tumour-no targeted) was conducted (Supplementary Figure 1). The only statistical significance pairwise Bonferroni-adjusted comparison was between other solid tumour-no targeted and breast-targeted (*p* = 0.003).

### Bayesian model

BMA was carried out (Table 2A) to determine the best model to explain the PFS2/PFS1 ratio. According to posterior probability, the best model is the one in which only treatment is included as a prognostic factor (model 1, 0.232). Moreover, the posterior probability that this variable is non-zero is 0.868, which is the highest of the independent variables. Therefore, the best four models include treatment type as a prognostic factor. Patients who received targeted therapy had higher log odds of having a PFS ratio >1.3 than patients receiving standard of care (posterior mean: 1.938, posterior standard deviation: 0.724, CI: 0.664–3.434). Convergence and effective sample size was within acceptable boundaries (Table 2B).

**Table 2:** (A) Model selection according to the posterior probability of model by Bayesian model averaging and (B) Bayesian logistic regression. Best model according to Bayesian model averaging.

(A)								
	p!=0	EV	SD	Model 1	Model 2	Model 3	Model 4	Model 5
Intercept	100	−1.175	2.053	−2.674	−0.527	−1.377	−3.602	1.084
Age	48.7	−0.028	0.035	–	−0.057	−0.063	–	−0.053
PS	6.0	−0.031	0.237	–	–	–	–	–
Treatment	86.8	1.644	0.921	1.847	1.904	1.897	1.943	–
Leucocytes	6.3	<0.001	<0.001	–	–	–	–	–
Lymphocytes	19.5	<0.001	<0.001	–	–	<0.001	<0.001	–
Neutrophils	6.1	<0.001	<0.001	–	–	–	–	–
RMH score	8.1	0.068	0.342	–	–	–	–	–
nVar				1	2	2	2	1
BIC				−311	−311	−309	−308	−308
Post prob				0.232	0.222	0.078	0.071	0.050

(B)									
Parameters	Mean	SD	2.5%	25%	50%	75%	97.5%	Rhat	N.eff
Intercept	−2.790	0.560	−4.009	−3.123	−2.749	−2.392	−1.871	1	900
Treatment (ref. no)	1.938	0.724	0.664	1.434	1.951	2.400	3.482	1	1000
Deviance	60.008	2.040	58.010	58.570	59.400	60.790	65.359	1	520

Models are ordered according to posterior probability; estimates are in the log odds scale.

Best mode includes only Treatment (No targeted therapy as reference) as an independent factor.

*p!=0* posterior probability that the variable is in the model, *EV* BMA posterior mean, *SD* posterior standard deviation, *nVar* number of variables in the model, *BIC* Bayesian Information Criterion, *post prob* the posterior probability of the model.

### Treatment beyond progression

Intriguingly, 53% of patients who had experienced PD during the experimental matched treatment were able to receive further treatments, while only 25% of patients treated with standard of care could be treated beyond progression. Although these differences are not statistically significant (*p* = 0.058), it should be underlined that receiving experimental matched molecular therapies does not reduce opportunities for further lines of treatment. Figure 4d shows the number of treatment lines received beyond progression in both patient groups.

## Discussion

We present here the results of a molecular-based approach using a customised panel at a single academic institution (Supplementary Table 4). This molecular screening was aimed at selecting the best potential therapeutic strategy for advanced cancer patients to receive treatment within our early clinical trial programme. Despite the small number of patients who presented actionable alterations (mutations, gene fusions, etc.), we demonstrated that this molecular profiling is feasible and effective. Our results confirm a substantial benefit in the molecularly matched cohort receiving experimental targeted therapies. The primary endpoint of increasing the PFS2/PFS1 ratio was reached and 33% of patients receiving molecular-matched therapies had a ratio ≥1.3, compared with only 7.9% of those who did not qualify for such therapies. Moreover, disease control was seen in 68.7% of patients treated in the experimental arm, while it was only observed in 36.4% of those assigned to standard care. It is worth mentioning that 53% of patients treated in early clinical trials received post-progression treatment vs only 25% of those treated with standard therapies. Utilising the window of opportunity with experimental targeted agents early on does not preclude receiving further lines of standard therapies. Conceivably, early treatment to block molecular drivers contributes towards better disease control and avoids rapid clinical deterioration, facilitating further therapeutic interventions.

Precision medicine for cancer patients has been recognised as a valuable strategy in solid tumours. Nevertheless, due to intrinsic features such as tumour heterogeneity, clonal evolution and individual resistance patterns, blocking a single molecular alteration is not always sufficient to control cancer growth and progression. Previous studies have evaluated the efficacy of using multigene molecular screening approaches to personalised cancer therapy, with controversial results. The SHIVA trial [16] could serve as a paradigm for what is still incompletely understood when applying precision medicine in cancer patients. In this trial, no benefit in median PFS was observed in molecularly oriented patients vs a conventional approach (hazard ratio = 0.88, *p* = 0.41), suggesting that off-label use of molecularly targeted agents does not improve PFS compared with standard treatment [15]. Several other prospective and retrospective clinical trials testing molecular-based approaches have been successively conducted, with contradictory positive and negative results. In the present non-randomised study, the PFS2/PFS1 ratio was chosen as a primary endpoint, as proposed in several previously published randomised trials [17, 18]. Assuming that PFS time shortens in inverse correlation with successive lines of treatment, we adopted the goal of a PFS2/PFS1 ratio ≥1.3 as a surrogate of clinical benefit. This was widely used as a sensible way to analyse the potential benefit obtained over a pre-treated population.

The base for an accurate molecular definition of solid tumours is pre-analytic evaluation. The assessment of test characteristics and quality assurance technical parameters as well as the estimation of the tumour cell fraction is a critical point for the interpretation of the molecular alterations [47]. In particular, low percentages of neoplastic cells are sometimes associated with unreliable results. Therefore, the percentage of tumour cells must be estimated either through microdissection technique or selection of block interest region, excluding necrotic region, normal tissue and the lymphocyte infiltration areas. In case microdissection is performed, higher sensitivity is obtained and more chance to detect a tumour-specific mutation [48].

Personalised panels are not ordinarily used in standard practice due to their inherent logistic complexity. The validation of our customised panel was carried out according to international guidelines [49] to ensure the quality of the results. Nonetheless, a salient feature of our molecular screening approach is its high plasticity. Our customised panel can be modified and adapted in line with new translational breakthroughs, keeping it updated with the latest oncology discoveries. Primarily, our panel was designed to target selective molecular alterations, which could facilitate patient inclusion into clinical trials. Despite being quite small, this customised panel allows us to evaluate the major known oncogenic drivers, including detection of possible concomitant molecular alterations, which is not possible when selecting single driver alteration detection devices. Moreover, using a multigene panel minimises the risk of enrolling patients presenting multiple drivers that could reduce the potential benefit of gene–drug matches. Clearly, selecting a targeted panel implies a limited number of genes. In our case, this was large enough to contain the most interesting molecular aberrations, yet with a medium size, simplifying tissue necessity and bioinformatic analysis, and reducing sample price. On the other hand, the choice of an in-house customised panel could have several limitations, most fundamentally the higher risk of reproducing important mistakes at some point during the process (panel design, results interpretation, etc.), which underscores the imperative necessity of using quality control of processes over the whole method.

A major challenge for oncologists nowadays is defining which genomic alterations are potentially suitable clinical targets. In order to advance the implementation of precision medicine in oncology common practice, several recent publications have tried to standardise genomic data reporting and interpretation in oncology. In our precision medicine group, we incorporated a weekly multidisciplinary institutional tumour board meeting to discuss each individual genomic result available. As previously stated, this board is formed by a team of experts in clinical oncology, molecular biology, pathology, clinical genetics and bioinformatics. Each result is discussed taking into account the ESMO Scale for Clinical Actionability of Molecular Targets [36]. In addition, we individually review specific genomic results to debate common issues such as interpreting mutations with low allele frequency, the importance of tumour content or the potential role of other concomitant mutations [50]. According to our final decisions, patients are stratified into eligible or not for targeted early clinical trials. We believe the high success rate obtained with our new agents could be attributable to the careful selection process carried out by our tumour board

Specifically, we included mainly patients whose tumours harboured PIK3CA mutations, FGFR or DNA repair alterations (non-BRCA). Patients were enrolled in early phases trials developing novel PIK3CA, AKT, FGFR, HER2, PARP inhibitors, Check-1 and other targets inhibitors, mostly with basket indications. This new concept of molecular-focused rather than origin-focused tumours has also contributed to revolutionising cancer care. Despite the important caveat that each driver could have a different role depending on cancer type, it seems possible that accurate molecular analysis and case discussion on a molecular board could improve the management of patients presenting with a potential therapeutic driver.

Assessment of DNA damage response genes is also very useful in precision medicine and could bring clinical benefit when patients with several tumour types are treated with novel agents, such as Check-1/2 inhibitors or novel PARP inhibitors. This strategy was highly beneficial in patients with non-gynaecological tumours, such as anal or small cell lung cancers.

This study presents several limitations potentially leading to uncontrolled biases, such as its retrospective and non-randomised nature, as well as the single-centre setting, which yielded a reduced patient sample. Another drawback could be the short follow-up, yet this could also limit the magnitude of benefit in the targeted therapy cohort. Indeed, at data cutoff (October 15, 2020), treatment is ongoing in 41% of patients included in the molecular-matched cohort, compared to only 9% in the standard therapy arm. Furthermore, these are early clinical trial results with a median follow-up time of >3 months, classically a key time point for phase I development. Focusing on patients who are still under treatment and had a prior line, however, all those in the standard therapy cohort (6/6) had a PFS2/PFS1 ratio <1.3, compared to only 37.5% (3/8) in the experimental treatment arm.

In conclusion, validating the true clinical relevance and actionability of each genomic event is critical and still far away from global standardisation. This highlights a need to incorporate a multidisciplinary molecular tumour board in academic institutions to expand the use of precision oncology. Patients with actionable molecular aberrations may benefit more from specific targeted therapies than from standard treatments. Precise selection of targeted agents matched to molecular alterations in advanced cancer patients may play a significant role in improving treatment decisions and management. One of the best ways to overcome some limitations would be the circulating tumour DNA analysis. The liquid biopsy could permit dynamic molecular profiling of all patients diagnosed with solid tumours improving a precision approach.

### Reporting summary

Further information on research design is available in the Nature Research Reporting Summary linked to this article.

## Supplementary information

Supplementary Material

Reporting Summary

## Data Availability

The datasets, including the redacted study protocol, redacted statistical analysis plan and individual participant’s data supporting the results reported in this article, will be available from the corresponding author on reasonable request.
