# Supplementary material for: Molecular profiling of advanced solid tumours. The impact of experimental molecular-matched therapies on cancer patient outcomes in early-phase trials: the MAST study
Source: Br J Cancer. 2021 Sep 7;125(9):1261–9. doi: 10.1038/s41416-021-01502-x (PMC8548537; doi:10.1038/s41416-021-01502-x)
Supplement: Supplementary file 1 — Supplementary Material [file 41416_2021_1502_MOESM1_ESM.docx]

Supplementary Figure 1: Progression-free survival (PFS2) by cancer type and treatment arm


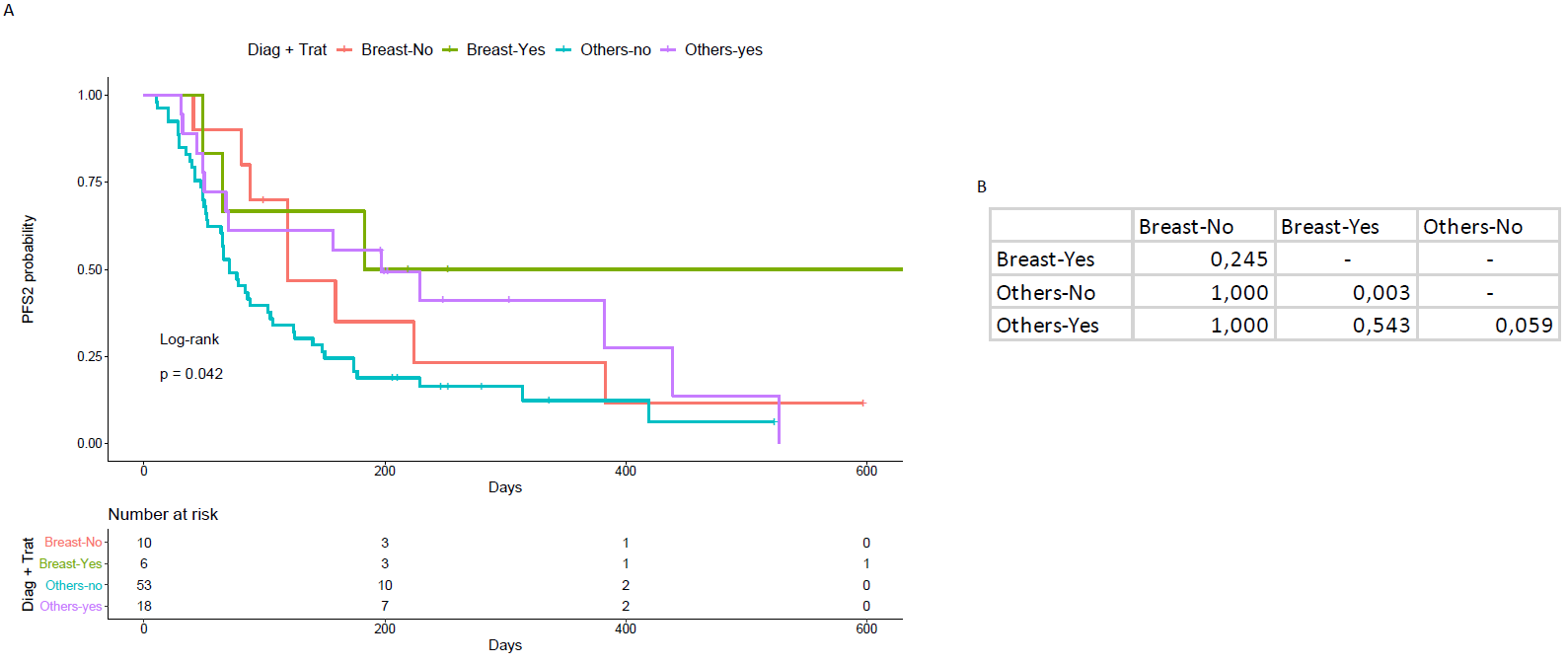


(A) Progression-free survival (PFS2) for breast-targeted therapy, breast-no targeted therapy, other solid tumor-targeted therapy, other solid tumor-no targeted (PFS2) (B) Bonferroni adjusted p value comparison subgroup analysis in the 4 different groups.

Supplementary Table 1: Experimental type of targeted drugs

| Target | Drug | Cancer type | N of patients | % |
| --- | --- | --- | --- | --- |
| PIK3CA | TKI | Breast | 8 | 25 |
| CHK1 | TKI | AC, SCC, Ovarian | 5 | 15.6 |
| MEK + PARPi | TKI | Ovarian | 4 | 12.5 |
| FGFR | TKI | Cholangiocarcinoma, Basal-cell carcinoma | 4 | 12.5 |
| MET | mAb | HNSLC, SCLC | 3 | 9.2 |
| PARPi | TKI | Ovarian, Breast | 3 | 9.2 |
| HER2 | TKI | Breast, Gynecological | 3 | 9.2 |
| AKT | TKI | Gynecological | 2 | 6.2 |
|  |  |  |  |  |

* AC: anal carcinoma, HNSCC: head and neck squamous cell carcinoma, mAb: monoclonal antibody, SCC, Squamous-cell carcinoma,TKI: Tyrosine kinase inhibitors

Supplementary Table 2: Cancer type, pathway alteration and type of experimental targeted agents used in treated patients

| Cancer Type | Pathway involved | Target drug |
| --- | --- | --- |
| Anal cancer | DDR pathway alterations | Chk1 Inhibitor |
| Anal cancer | DDR pathway alterations | Chk1 Inhibitor |
| Basal-cell carcinoma | FGFR2 mutation | FGFR inhibitor |
| Biliary tract cancer | FGFR2-BCC1 fusion | FGFR inhibitor |
| Biliary tract cancer | FGFR2-BCC1 fusion | FGFR inhibitor |
| Biliary tract cancer | FGFR2-BCC1 fusion | FGFR inhibitor |
| Breast cancer | PIK3CA mutation | PI3Kα-specific inhibitor |
| Breast cancer | PIK3CA mutation | PI3Kα-specific inhibitor |
| Breast cancer | PIK3CA mutation | PI3Kα-specific inhibitor |
| Breast cancer | PIK3CA mutation | PI3Kα-specific inhibitor |
| Breast cancer | PIK3CA mutation | PI3Kα-specific inhibitor |
| Breast cancer | PIK3CA mutation | PI3Kα-specific inhibitor |
| Breast cancer | PIK3CA mutation | PI3Kα-specific inhibitor |
| Breast cancer | PIK3CA mutation | PI3Kα-specific inhibitor |
| Breast cancer | PIK3CA mutation | AKT inhibitor |
| Breast cancer | HER2 mutation | pan-HER tyrosine kinase inhibitor |
| Breast cancer | HER2 mutation | pan-HER tyrosine kinase inhibitor |
| Breast cancer | DDR pathway alterations | Chk1 inhibitor |
| Endometrial carcinoma | PTEN mutation | AKT inhibitor |
| HNSCC | MET pathway alteration | RTK inhibitor |
| SCC | DDR pathway alterations | Chk1 Inhibitor |
| SCC | MET pathway alteration | RTK inhibitor |
| SCC | MET pathway alteration | RTK inhibitor |
| Ovarian carcinoma | DDR pathway alterations | Chk1 Inhibitor |
| Ovarian carcinoma | DDR pathway alterations | Chk1 Inhibitor |
| Ovarian carcinoma | DDR pathway alterations | Chk1 inhibitor |
| Ovarian carcinoma | DDR pathway alterations | Chk1 inhibitor |
| Ovarian carcinoma | DDR pathway alterations | PARP inhibitor + MEK inhibitor |
| Ovarian carcinoma | DDR pathway alterations | PARP inhibitor + MEK inhibitor |
| Ovarian carcinoma | DDR pathway alterations | PARP inhibitor + MEK inhibitor |
| Ovarian carcinoma | DDR pathway alterations | PARP inhibitor + MEK inhibitor |
| Ovarian carcinoma | HER4 mutation | pan-HER tyrosine kinase inhibitor |

DDR: DNA damage response, HNSCC: head and neck squamous cell carcinoma, PARP: poly(ADP-ribose) polymerase, RTK: receptor tyrosine kinases, SCC: Squamous-cell carcinoma. Each row represents a single patient.

Supplementary Table 3: Multivariable Cox-proportional hazard regression

| Variable | | | Hazard Ratio | CI 95% | Pr (>\| z \|) |
| --- | --- | --- | --- | --- | --- |
| Treatment type | | Standard vs | . | . | . |
|  |  | Targeted Therapy | 0.478 | 0.257 – 0.889 | 0.020 |
| PS | | PS0 | . | . | . |
|  |  | PS1 | 3.237 | 1.350 – 7.760 | 0.008 |
|  |  | PS2 | 8.487 | 2.782 – 25.892 | <0.001 |
| Leukocytes | | | 1.0002 | 1.0001 – 1.0003 | <0.001 |
| RMH prognostic score | Good | | . | . | . |
|  | Poor | | 1.821 | 1.010 – 3.2.283 | 0.046 |
| Lymphocytes | | | 0.9996 | 0.9992 – 0.9999 | 0.017 |
| Events / N | | | 76 / 87 | | |
| AIC* | | | 484.87 | | |
| Proportional hazards assumption | | | Global P = 0.49 | | |

Result of stepwise (forward-backward) analysis based on AIC with age, treatment type, previous treatment (yes or no), performance status (PS), leukocyte, lymphocytes, neutrophil, Royal Marsden Hospital (RMH) Prognostic Score and the interaction between previous treatment and treatment type as independent factors. *AIC: Akaike information criterion.

Supplementary Table 4: Customized gene panel
